# Bilateral changes in afterhyperpolarization duration of spinal motoneurones in post-stroke patients

**DOI:** 10.1371/journal.pone.0189845

**Published:** 2018-01-16

**Authors:** Bożenna Kuraszkiewicz, Jia-Jin Jason Chen, Hanna Goszczyńska, Yu-Lin Wang, Maria Piotrkiewicz

**Affiliations:** 1 Nalecz Institute of Biocybernetics and Biomedical Engineering, Polish Academy of Sciences, Warsaw, Poland; 2 Institute of Biomedical Engineering, National Cheng Kung University, Tainan, Taiwan; 3 Department of Rehabilitation, Chi Mei Medical Center, Tainan, Taiwan; 4 Center for General Education, Southern Taiwan University of Science and Technology, Tainan, Taiwan; Szegedi Tudomanyegyetem, HUNGARY

## Abstract

This paper extends the observations presented in the previously published work on the afterhyperpolarization (AHP) duration changes in motoneurones (MNs) on the paretic (more affected) side of 11 post-stroke patients by the same analysis on the non-paretic (less-affected) side. The estimated AHP duration for patients’ MNs supplying more-affected muscles was significantly longer than control values and the elongation decreased with patient age and disorder duration. For MNs supplying less-affected muscles, dependency of AHP duration on age was closer to the control data, but the scatter was substantially bigger. However, the AHP duration estimate of less-affected MNs tended to be longer than that of controls in the short time elapsed since the stroke, and shorter than controls in the long time. Our results thus suggest that the spinal MNs on both sides respond to the cerebral stroke rapidly with prolongation of AHP duration, which tends to normalize with time, in line with functional recovery. This suggestion is in concert with the published research on post-stroke changes in brain hemispheres. To our knowledge, these dependencies have never been investigated before. Since the number of our data was limited, the observed trends should be verified in a larger sample of patients and such a verification could take into account the suggestions for data analysis that we provide in this paper. Our data are in line with the earlier published research on MN firing characteristics post-stroke and support the conclusion that the MUs of the muscles at the non-paretic side are also affected and cannot be considered a suitable control for the MUs on the paretic side.

## Introduction

Stroke is a devastating condition often resulting in spastic hemiparesis. In many clinical studies, the non-paretic side is considered to be “healthy” and therefore the common practice is to compare results between paretic and non-paretic extremities (e.g. [1, 2]). However, it has been shown [3] that the stroke lesion at one side of the brain alters neuronal activity in both affected and contralateral hemispheres. Moreover, the intact hemisphere contributes to functional recovery after stroke. Thus, it cannot be expected that the spinal motoneurones (MNs) and muscles on the non-paretic side will remain unaffected after stroke.

Indeed, in a recently published paper, McNulty et al. [4] have shown that the discharge rates of the MNs supplying muscles on the non-paretic side were significantly higher not only from those on paretic side, but also from control MNs. This finding is in line with the above description of brain connectivity changes after stroke lesions. Therefore, the authors concluded that the non-paretic side does not provide a valid control for motor unit activity on the paretic side and that control data should always be recorded in healthy subjects.

The range of discharge rates of a MN is related to its afterhyperpolarization (AHP) duration. The AHP duration in MNs of stroke patients was estimated by [2] and [5]. It has been observed in both studies that AHP duration estimates measured in MNs supplying the paretic side were significantly longer than those measured on patient’s non-paretic side. Suresh et al. [5] did not find significant differences in AHP duration estimates between control MNs and those on non-paretic side, although mean AHP duration values on the patient’s non-paretic side were slightly shorter.

The goal of the present paper was to analyze the bilateral changes in motor control of stroke survivors by comparing AHP duration from both sides with those recorded in a control group. This analysis was enriched by comparing the influence of age and disorder duration on AHP estimates between subject groups, which to our best knowledge has never been done earlier. Our results confirm the findings of [4], that muscles and MNs on both sides are affected, although in a distinct manner. Therefore, we will be using the terms *less*- and *more-affected* instead of *non-affected* and *affected*, respectively.

## Materials and methods

The methods applied in this study were described in our previous paper [6]. Here, only the most important details will be given.

### Subjects

The study population included 11 post-stroke patients, aged 30–77 years, all suffering from spasticity and seeking BOTOX treatment. The patients were recruited from the Department of Rehabilitation in Chi-Mei Hospital, Tainan, Taiwan. The patients’ characteristics are collected in Table 1. The control group comprised eight subjects aged 22–64 years. None of the control subjects had any history of a neuromuscular disorder. All control subjects and patients were right-handed. The experiments were performed in agreement with the declaration of Helsinki. Each subject gave written informed consent to the experimental procedures. The study was approved by the Ethical Committee at Chi-Mei Hospital, Tainan.

**Table 1:** Patient characteristics.

Patient's code	Age (years)	Affected side	Disease duration (years)	Brunnstrom stage
S01	71.38	left	12.33	4
S02	48.54	left	12.25	3
S03	30.25	right	12.17	3–4
S04	48.33	right	12.08	2–3
S05	53.58	right	11.67	3–4
S06	40.00	right	1.33	3
S07	41.75	left	1.94	3–4
S08	40.25	right	0.90	3
S09	56.58	left	0.67	n.a.
S10	76.83	right	8.00	n.a.
S11	47.25	right	1.25	n.a.

### Experiments

Subjects lay down comfortably on a bed so the arm was slightly abducted. The subject was provided with auditory feedback of the motor unit (MU) discharges and was instructed to perform minimal isometric contraction for three minutes and then slowly increase the contraction force during fourth and fifth minute, until roughly 50% maximum voluntary contraction was achieved.

An experienced doctor collected the MU potentials from the brachial biceps, from both sides in patients and from the left side in control subjects. The signals were recorded by a bipolar concentric electrode mounted in a needle used for BOTOX injections, amplified by a Medelec Premiere Plus electromyograph (Vickers Medicals, Woking, UK), sampled at 10 kHz by an A/D converter with 12-bit resolution, and stored on a laptop computer for off-line analysis. Software for data acquisition was developed by means of LabView environment.

### Data analysis

For all stages of data analysis custom software was developed.

Typically, the potentials of several MUs that were simultaneously active were recorded in each experiment. MU recordings were decomposed into constituent single MU potential trains by an operator-computer interactive method described in [7]. After the preliminary automatic computer identification, the results were verified by an experienced human operator who corrected the misclassifications.

Single MU potential trains were subjected to variability analysis based on the method introduced by the pioneering work of Tokizane and Shimazu [8]. They have noticed that the typical relationship of the standard deviation of interspike intervals (ISIs) on their mean value (*s-τ* curve) is composed of two distinct short- and long-interval fragments. Subsequently, Person and Kudina hypothesized that the transition between these fragments may be related to the MN AHP duration [9]. Indeed, it has been shown by computer simulations [10, 11], in direct measurements from cat MNs [12] and in human experiments [13] that the transition interval (*TI*) delimiting these two fragments is correlated with the AHP duration for each single MN. Consequently, this method was applied in the Warsaw lab for comparative studies of AHP duration [14–16].

**Fig 1:**
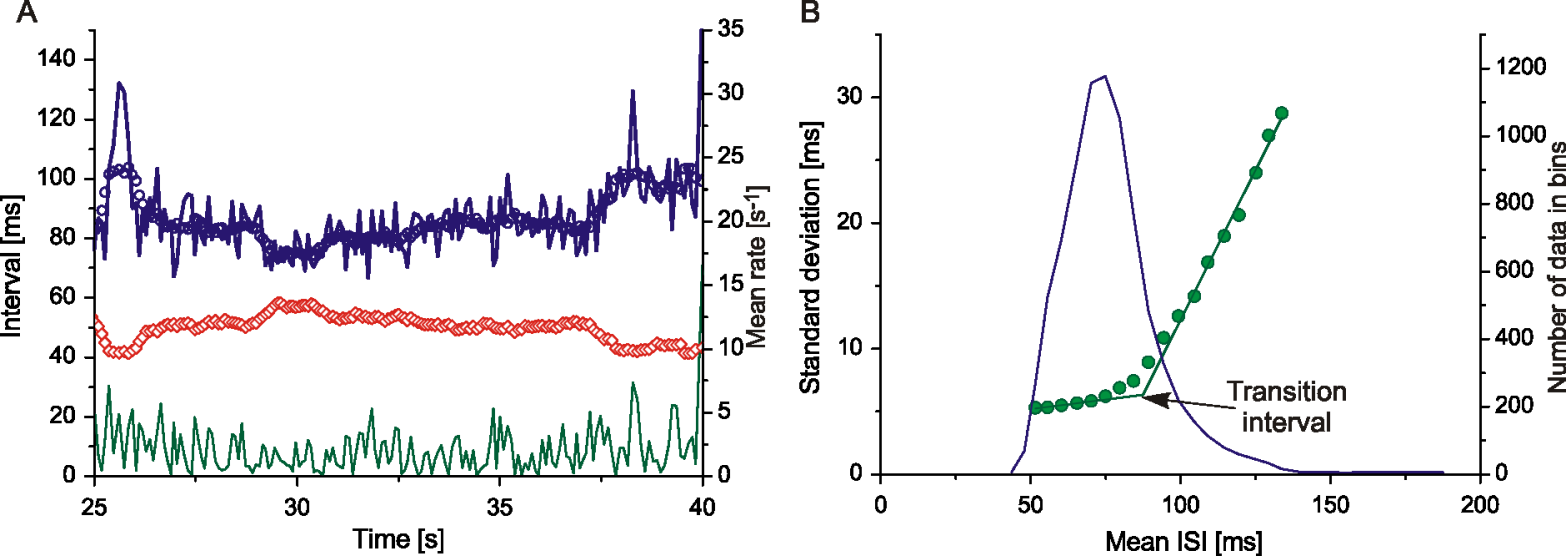
AHP duration estimation. A) an example of data from a control subject: blue line, interspike intervals, blue circles, their moving average, orange diamonds, mean firing rate (inverse of ISI moving average), green line, absolute value of consecutive difference, all plotted vs. time. Note different scale for firing rate (right axis); B) green circles, SD(MISI) plot, lines fitted to the linear fragments of the plot. Their intersection determines the transition interval, which is an estimate of AHP duration (arrow); blue line, number of data in bins, right axis.

Standard deviation should be calculated from the long stationary fragments of MU potential recording (at least 50 consecutive potentials per experimental point). These are not always available, especially in patients, so we have changed this method of variability analysis to another, which was applicable to the non-stationary recordings. This method was adopted from Holt et al. [17] and described in detail in [14]. It uses the absolute value of consecutive difference between two adjacent ISIs, which is plotted vs. the mean ISI calculated from the same two intervals. Such a plot is a cloud without apparent structure, which is not shown here. To estimate the AHP duration, the SD and mean ISI (MISI) was calculated from consecutive difference values in 10 ms bins, moved every 5 ms. Fig 1A shows an example of data from a control subject: subsequent ISIs as the function of time along with their moving average, firing rate and consecutive difference. Fig 1B presents SD plotted against MISI. The peripheral SD values calculated from insufficient number of points are neglected. The intersection of two lines fitted to the linear fragments of the SD(MISI) plot determines the value of the TI. The obtained SD(MISI) relationships are equivalent to the Tokizane and Shimazu s-τ curve with respect to the TI estimation [14], which was confirmed by computer simulations based on our threshold-crossing model [10, 11].

Statistical analysis of data was performed using Mann-Whitney test to compare data for disease duration and T-test for independent samples for remaining comparisons. The Pearson regression tool was applied for analysis of dependencies. The correlation coefficients were assessed and compared using Student’s *t* test considerations and Fischer’s *r*-to-*z* transformation. The analysis was carried out using STATISTICA (StatSoft®, ver. 10) and Microsoft Excel 2017. A p-value of < 0.05 was considered statistically significant.

## Results

Altogether, 39 MUs were recorded from control subjects and 113 from patients: 64 and 49 from more- and less-affected muscles, respectively. The patients’ MU firing rates were in the range 4.6–14.3 s^–1^ (mean 7.9 s^–1^) for more-affected muscles and 5.3–16.9 s^–1^ (mean 9.6 s^-1^) for less-affected muscles. For control subjects the range of MU firing rates was 5.4–20 s^–1^ (mean 10.6 s^–1^). The differences were statistically significant (p<10^−4^) when both less- affected and control MUs were compared with more-affected MU data, but not significant for comparison between less-affected and control MUs. Patients’ MU firing was often irregular and it was not always possible to follow single MUs through the entire experiment, presumably due to fatigue. Therefore, after decomposition, some MUs could not be analyzed for variability because of an insufficient number of points.

Complete SD(MISI) relationships were obtained for 25 control MUs and 65 patient MUs (37 and 28 from more- and less-affected muscles, respectively). The TIs for more-affected MNs (mean 135.21. SD 12.049) were significantly longer from those less-affected (mean 113.62. SD 22.54. p = 0.011) and control MNs (mean 97.22. SD 13.28. p = 7.2*10^−6^). The difference between TIs for control and less-affected MNs was also significant (p = 0.041).

**Fig 2:**
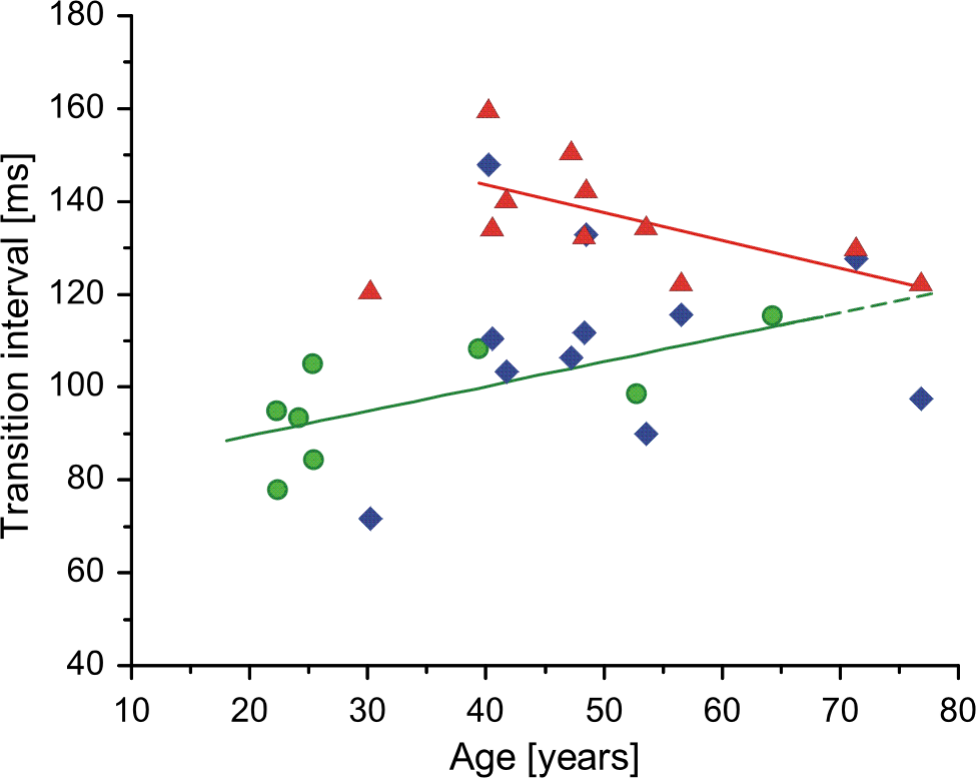
Relationship between mean values of TI, calculated for each subject from single MU data, and subject’s age: Circles, control subjects; triangles, patient’s more-affected muscles; diamonds, patient’s less-affected muscles. Note that data for older patients are close to the control regression line. Note also an outlier for the youngest patient, who had the stroke 12 years before the experiment.

In Fig 2 the mean values of TIs (calculated for each subject from all MUs analyzed) are plotted vs. age for control subjects and patients. It can be clearly seen that the AHP duration estimate of more-affected MNs is substantially increased as compared to control data for younger patients, and that it tends to converge with control data for older patients.

The regressions were significant for data from both control (R = 0.692, p = 0.028) and more-affected MUs (R = 0.699, p = 0.026, calculated without the outlier). Also the difference between both regression lines was highly significant (p = 0.003). The regression analysis was not performed for data from less-affected MUs, since their big scatter raised no doubts that this regression would be highly insignificant.

In our previous study [6], which concentrated only on the patients’ more-affected MNs, we observed that both age and disease duration contribute to the observed decrease in estimated AHP duration. To visualize the dependency from the disease duration, in the next plot we transformed the data by calculating the distance of each patient’s TI from the regression line:

$$\Delta TI\left(d_{d}\right)=TI\left(d_{d}\right)–a*d_{d}–b,$$ (1)

where *d*_*d*_ is the disorder duration [yrs], *a* = 0.53 ms/yrs. *b* = 78.952 ms (coefficients of control regression line). We also excluded from statistical analysis the data of patients older than 55 years, since their TIs were close enough to the control line due to the dependency of this characteristic on age.

**Fig 3:**
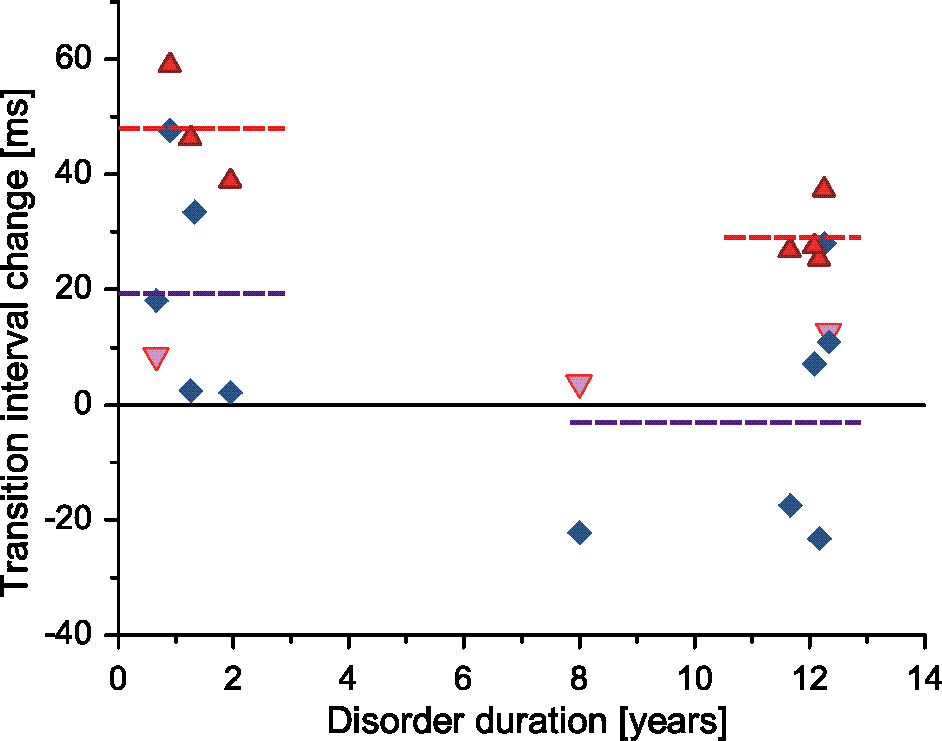
Distance ΔTI for less- and more-affected MNs, plotted vs. disorder duration. Explanation of symbols as in Fig 2, except for 3 outliers of more-affected MNs for patients older than 55 years (triangles down). Dashed horizontal lines indicate mean values of Δ*TI* for short and long time after stroke.

Here, we repeated this analysis also for less-affected MNs (Fig 3).

The time after stroke contains two distinct clusters: short (around 1 year) and long (around 12 years), with only one value in between (8 years). The Man-Whitney test was applied to compare data between clusters and more- and less-affected MNs within each cluster (the data from more-affected side of patients older than 55 years were excluded). The data of more-affected MNs were significantly different from those of less-affected ones in both clusters. The comparison of data from more-affected MNs yielded p value slightly exceeding the imposed limit (p = 0.0518), with the mean values of 48.07 and 29.26 ms for short and long time after stroke, respectively. For less-affected MNs this difference was insignificant. Note, however, that the mean value of the TI of less-affected MNs is higher than control TI (18.5 ms) for the short, and lower than control TI (-2.8 ms) for the longer post-stroke time.

## Discussion

This paper is the third in a series of recently published studies, in which firing characteristics of MNs supplying muscles on both paretic and non-paretic sides were compared in post stroke patients. The results of all three papers confirm that less-affected MNs differ from control ones. Nevertheless, there are certain discordances concerning observed firing rates. The study of Suresh et al. [18], reported significant differences in firing rates between more- and less-affected MNs and between those more-affected and controls, but not between less-affected and control MNs. The same result was obtained in our study, whereas McNulty et al. [4] found firing rates of less-affected MNs significantly different from those more-affected and controls, but not between controls and more-affected MNs. In our study the highest firing rates were recorded from control MNs, whereas in the other two studies, the highest firing rates were seen in less-affected MNs. However, the differences between groups were within 1–2 p/s, so the discordances might be expected, given that the number of subjects differed between studies (patients/controls: 28/16 in [4], 11/7 in [18], 11/8 in the present study), and that considerable inter-individual scatter of the results was apparent in all studies.

The number of subjects in our study and that of Suresh et al. [18] was low. It might have been acceptable in [18], where only statistical analysis of group differences was performed, but was certainly not sufficient to draw firm conclusions on time-dependencies in our study. Nevertheless, we decided to complete this analysis assuming that taking time factor into account would enrich our study, even though we can only indicate the tendencies, which need verification in a larger sample of patients. Moreover, the factors of age and disease duration were indicated as important for studies of brain activity post-stroke [3].

Nevertheless, the limited patient’s sample size have been beneficial for the present study. If it had been much larger, patient’s age and disorder duration would have had distribution close to normal. This would have resulted in the age dependency of the cloud shape, where it would have been hardly possible to observe any trends or outliers. Another fortunate circumstance was that our youngest patient suffered the stroke at the age of 19 years, which resulted in the clear outlier in Fig 2, suggesting that disorder duration might be of importance. Our patient sample was unique also because we had 4 young patients with post-stroke time not exceeding 2 years, whose AHP durations were visibly longer from other patients. Given that the difference between patients with short and long disorder duration did not reach the level of statistical significance, we presumably could have not noticed the tendency illustrated by Fig 3 if this patient subgroup had not been present.

Our results indicate that the AHP duration of MNs supplying both more- and less-affected muscles tends to increase soon after stroke and gradually returns to control values thereafter. This corresponds to the description of changes in brain activity after stroke. In the first few days after stroke, the ischemic lesion is followed by reduced brain activity in the affected hemisphere and there is a gradual increase in activity thereafter consistent with functional recovery in both hemispheres [3].

The question is: which mechanisms may induce the observed changes in AHP duration of MNs? The early loss of corticospinal connections would certainly result in reduced synaptic inflow and its variability (synaptic noise). The inflow variability is directly related to interspike interval (ISI) variability, which our method for estimation of AHP duration relies on. It might be possible that the decreased synaptic noise prolongs ISIs, resulting in lower firing rates and longer AHP duration estimates. There is evidence supporting decreased variability in more-affected MNs [2, 5, 19, 20]. In our previous study [6] we also observed decreased ISI variability in patients’ more-affected MNs, when compared in the short-interval range, although the difference was not significant. In any case, the possible contribution of this factor deserves future elucidation.

Suresh et al. [18] suggested that increases in AHP duration might result from changes in activity of specific neuromodulator pathways following stroke. The neuromodulators are known to decrease AHP amplitude and enhance synaptic noise (e.g. [21]). Therefore, reduced supply of neuromodulators might result in the prolongation of estimated AHP duration in a manner similar to that explained above. However, it still remains to be proved that in healthy subjects neuromodulators are active in the voluntary isometric contractions of low force, i.e. those, at which AHP duration is estimated.

Animal studies have shown that many biochemical and cellular processes are triggered by stroke within minutes and hours, as e.g. activation of immunomodulators and enhanced expression of neuroprotective proteins, nerve growth factors, and neurotransmitter receptors. Such processes participate in the formation of new synapses and the sprouting of axons. Additionally, new connections with MNs are developed to compensate for the lesion-induced loss of corticospinal fibres [22]. Whether and how these processes affect spinal MNs, remains to be elucidated.

It is also not clear how the described changes in brain activity may influence spinal MNs on the non-paretic side. As already mentioned, the contralesional hemisphere participates in functional recovery post-stroke. However, the exact functional role it plays during recovery seems to be complex (see [3] for details). This complexity may be partly responsible for the huge scatter of data collected from the less-affected MNs (e.g. Fig 2). This scatter may also reflect differences in patient’s lifestyle, e.g. mobility or need to regain self-sufficiency. If we assume that the decrease in AHP duration in less-affected MNs indicates their compensation of MN slowing on the paretic side, then the shorter AHP would indicate more intense activity of less-affected muscles. Moreover, the MN AHP duration post-stroke may depend on the speed of recovery [2] and on the efficacy of the rehabilitation process. This may be the explanation why we did not observe higher mean firing rates and shorter TIs at the non-paretic side as compared with control values, when pooled data was analyzed (although we did find shorter than normal AHP on average in the less-affected MNs in patients with a longer time after stroke, Fig 3). We have reason to think that many patients participating in the study [4] were recruited from the rehabilitation clinic. It is thus possible that the percentage of patients with a longer time past stroke and/or better recovery was higher in [4] than in our study, which would explain this discordance.

## Conclusions

In summary, our results support the conclusion of McNulty et al. [4] that the MUs of the muscles at the non-paretic side post-stroke are also affected and cannot be considered a suitable control for the MUs on the paretic side.

Our data indicate the possibility that spinal MNs respond to the cerebral stroke with prolongation of AHP duration, which tends to recover after the accident. These changes occur in parallel in more- and less-affected muscles.

Although the number of subjects participating in our study was small and not all observed relationships were statistically significant, the results may be helpful in indicating the directions of further research, which could verify our observations. This may be accomplished in several ways, including re-analysis of previously recorded data (e.g. [2, 4, 5]. For this re-analysis, the data should be sorted by patient’s age and by disorder duration. The factor of disorder duration should be studied in different age groups, and the factor of age, in different disorder duration groups. It would be also very interesting to investigate the influence of rehabilitation on the AHP duration on paretic and non-paretic sides.

This type of analysis may be important for further studies of changes in brain activity triggered by stroke. Grefkes and Fink [3] state that “…Most likely, time since stroke, severity of deficit at baseline, lesion size, location, and other biological factors (e.g. age of the patient) all contribute to interindividual differences”. AHP duration estimation might complement connectivity studies of the human brain and might be helpful to establish a reliable estimation of network disturbances at a single-subject level.

## Supporting information

S1 File The previously published article *Changes in Spinal Motoneuron “Fastness” in Post-stroke Spastic Patients*, related to the main topic. (PDF)

## Abbreviations

AHP: afterhyperpolarization
ISI: interspike interval
MISI: mean interspike interval
MN: motoneuron
MU: motor unit
SD: standard deviation
TI: transition interval
