# Supplementary material for: Bilateral changes in afterhyperpolarization duration of spinal motoneurones in post-stroke patients
Source: PLoS One. 2018 Jan 16;13(1):e0189845. doi: 10.1371/journal.pone.0189845 (PMC5770035; doi:10.1371/journal.pone.0189845)
Supplement: S1 File — (PDF) [file pone.0189845.s001.pdf]

Changes in Spinal Motoneuron "Fastness" in Post-stroke Spastic Patients

Ling-Yin Liang[1,2] Jia-Jin Jason Chen[2] Yu-Lin Wang[3]

Michal Jakubiec[4] Jolanta Mierzejewska[4] Maria Piotrkiewicz[4,*]

[1]Biomechanics and Movement Science Program, University of Delaware, Newark, DE 19716, USA
[2]Institute of Biomedical Engineering, National Cheng Kung University, Tainan 701, Taiwan
[3]Department of Rehabilitation, Chi-Mei Hospital, Tainan 710, Taiwan
[4]Institute of Biocybernetics and Biomedical Engineering, Polish Academy of Sciences, Warsaw 02-109, Poland

Received 6 Jul 2009; Accepted 5 Jan 2010

Abstract

Motor unit (MU) action potentials trains were recorded from brachial biceps of 11 post-stroke patients (Brunstrom stage 2–4) and 8 healthy volunteers. The variability analysis of interspike intervals (ISIs), aimed at the comparison of the duration of afterhyperpolarization (AHP) in motoneuron (MN), was performed on MUs of 39 controls and 64 patients. Our results concerning MU discharge characteristics conformed in general to the previous reports, showing positive serial correlation coefficients and lower MU firing rates in patients as compared with control subjects. We have found, however, that the positive correlation coefficients result from trends in MU firing rates related to less efficient control of the muscle force. ISI variability determined in short-interval range was the same in patients and control subjects. The transition intervals of variability-mean ISI characteristics, which were previously shown to correlate with AHP duration, were significantly longer in patients, but the prolongation decreased with patient's age and disease duration. Our results indicate that the spinal MNs respond to the cerebral stroke with prolongation of AHP duration, which tends to recover after the accident. These changes are less pronounced in older patients, presumably due to decreased MN plasticity. We conclude that the match between MN and muscle properties is preserved after stroke.

Keywords: Stroke, Age-dependence, Afterhyperpolarization (AHP), Human motoneuron (MN) firing patterns, Neuronal plasticity

1. Introduction

Spasticity is one of the consequences of central nervous system (CNS) lesions, which can result in serious problems for affected individuals and therefore is still a challenge to clinicians and scientists. Although considerable scientific and medical literature discusses the etiology and treatment of spasticity, the underlying mechanisms are largely unknown. In particular, motor unit (MU) firing patterns were often studied in spastic patients [1-7] but there is very little knowledge about the alterations which spastic disorders induced in motoneurons (MNs).

Spasticity after cerebral lesions, including stroke, is often thought to be connected with increased contraction of the skeletal muscle. Experimentally, such conditions may be reproduced with chronic electrical stimulation, which was investigated in several experiments [8-11]. The stimulation caused muscle phenotype shift from fast towards slow. In line with these results are reductions of firing rates observed in spastic post-stroke patients [2-6]. In contrast, spasticity after spinal cord injury (SCI) is more often associated with the chronic decreased use of the muscle, which leads to the muscle transformation towards faster phenotype, both in animals [12] and in humans [13]. In rat SCI model, this transformation in soleus muscle was shown to be accompanied by the decrease in motoneuronal afterhyperpolarization (AHP) duration [14]. However, MUs in other muscles and other species became slower after SCI [15,16]. Even single MUs in the given muscle could be differentially affected during long-term spastic hemiplegia [17].

The animal models of stroke are still under development. To our knowledge, studies on changes in motoneuron properties after stroke were not yet reported. It was suggested that the decreased MU firing rates could be due to the increased AHP duration of their MNs [18] and the match between motoneuron and muscle fiber characteristics in stroke patients is not preserved, which might reduce the efficiency of muscle contraction [4]. This issue thus calls for further investigations.

* Corresponding author: Maria Piotrkiewicz
Tel: +48-6599143 ext. 412; Fax: +48-6597030
E-mail: maria.piotrkiewicz@ibib.waw.pl

This paper presents the preliminary results of motoneuron "fastness" estimation in poststroke patients. The method was based on the analysis of the variability of interspike intervals (ISIs), which has been shown to correlate with the duration of AHP. Recently, it has been reported that AHP duration depends on the age of the subject [19]. The dependency between patients with post-stroke spasticity and control subjects is compared in this study.

2. Methods

Eleven post-stroke patients, aged 30–77 years, all suffering from spasticity and seeking BOTOX treatment, participated in the study. The patients were recruited from the Department of Rehabilitation in Chi-Mei Hospital, Tainan, Taiwan. The control group consisted of 8 Taiwanese subjects aged 22–64 years. None of the control subjects had any record of a neuromuscular disease. The experiments were performed in agreement with the Declaration of Helsinki. Each subject gave informed consent to the experimental procedures. The procedures were approved by the Ethical Committee at Chi-Mei Hospital, Tainan, Taiwan.

2.1 Experiments

During an experiment, the subject was lying down comfortably on a bed with arm slightly abducted. The subject was provided with auditory feedback of the MU discharges and was instructed to perform minimal isometric contraction for 3 minutes and then slowly increase the contraction force during the 4th and 5th minutes until roughly 50% maximum voluntary contraction was reached.

The electromyographic (EMG) data were collected from the brachial biceps (BB) by an experienced doctor. The potentials of single MUs were recorded by a bipolar concentric electrode mounted in a needle used for BOTOX injection. EMG signals were amplified by a Medelec Premiere Plus electromyograph (Vickers Medicals, Woking, UK), sampled at 10 kHz by an analog-to-digital converter with 12-bit resolution, and sent to a laptop computer for off-line analysis. Programs for data acquisition were constructed using LabView environment.

2.2 Data analysis

Specialized software was developed for the off-line data analysis. Potentials of several MUs that were active simultaneously were recorded in each experiment (Fig. 1). MU recordings were decomposed into constituent single MU potential trains by an operator-computer interactive method described in detail in [20]. The results of the preliminary computer identification were verified by an experienced human operator who corrected the misclassifications.

The variability analysis was based on the method introduced by the pioneering work of Tokizane and Shimazu [21], who noticed that the typical relationship of the standard deviation of ISIs on their mean value (s-τ curve) was composed of two distinct short- and long-interval parts. The authors got these relationships for a number of human muscles and for almost every one of them found two branches, which they interpreted as the manifestation of two MU types, tonic and kinetic. This study has received considerable interest, and the method has been applied in numerous studies [22-26], but the bifurcation of s-τ curves has never been confirmed. Instead, Person and Kudina proposed a hypothesis that the transition between short- and long-interval fragments of s-τ curve may be related to the motoneuron AHP duration [26,27]. Indeed, it has been shown by computer simulations [28,29], in human experiments [30] and in direct measurements from cat MNs [31] that the transition interval (TI) delimiting these two parts is correlated with the AHP duration for each single MN, although it is shorter than the AHP. Thus, the analysis of ISI variability allows for comparison of MNs with respect to their "fastness".

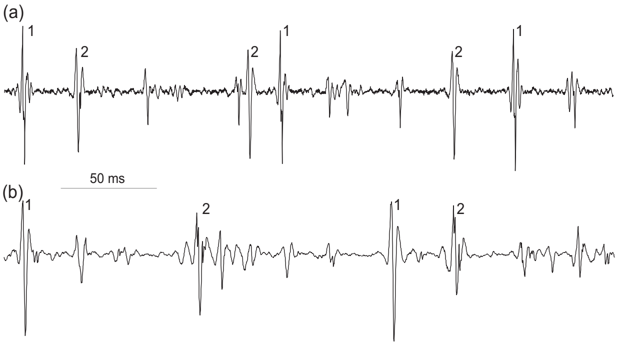

Figure 1. Examples of MU potential recordings: (a) control subject, (b) spastic patient. Potentials from the same MU marked by the same numerals.

Standard deviation (SD) should be calculated from long stationary fragments of the recording (at least 50 consecutive potentials per experimental point). These are not always available, especially in patients. To get a complete SD-mean plot, shorter periods of stationary discharge with similar mean ISI and SD need to be joined together. Such a procedure requires imposing joining criteria, which are disputable. Therefore, we changed this method of variability analysis to another, adopted from Holt et al. [32] and described in detail in [33]. Instead of SD, the latter method uses consecutive difference between two adjacent ISIs (*CD2*), which is related to the mean ISI calculated from the same two intervals (*MISI2*). As the result, a cloud of points is produced from each MU's data and getting the relationship, which is comparable with the s-τ curve, requires averaging *CD2* values falling into the vertical bins. The so-obtained *CDM*(*MISI*) relationship is equivalent to the s-τ curve with respect to the TI estimation, which was shown by the computer simulations based on our threshold-crossing model [28,29].

The complete *CDM*(*MISI*) plot for a MU usually cannot be obtained from less than 1000 *CD2*(*MISI2*) points. On most such plots, linear sections in short- and long-interval range could be distinguished by visual inspection. For each of these plots, the TI value delimiting short- and long-interval sections

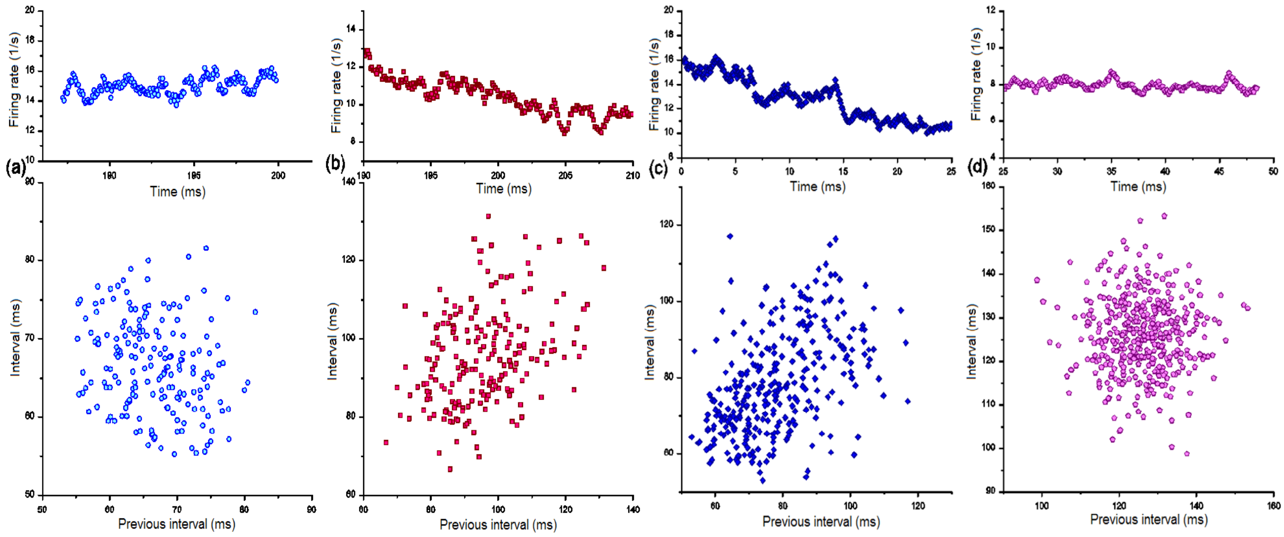

Figure 2. Examples of MU firing characteristics for control (left column) and spastic (right column) subject groups. (a) and (b): typical; (c) and (d): atypical characteristics. Upper panels, instantaneous firing rate time course; lower panels, joint interval histograms.

were estimated in order to quantify the position of the plot with respect to the ISI axis. If the transition between ranges was curvilinear, the limits of transition ISI range were determined by fitting straight lines to the initial and final parts of the plot (indicated by an operator) and the TI was set at the intersection of these lines. For the plots without visible curvilinear section, the data were divided into two subsets with division point set close to the shortest mean interval so that the short-interval subset contained 4 points. Then straight lines were fitted by means of the least-squares method to each subset; the division point was moved towards longer intervals with the step of 0.5 ms and this value, which corresponded to the total least squares sum, was taken as the TI.

2.3 Statistics

The statistical analysis of data was performed by the tools built into the program STATISTICA (StatSoft, ver. 6). The significance of differences was analysed by Student's paired *t*-test and the regressions by Spearman rank order correlations.

3. Results

Altogether, 39 MUs were recorded from the control subjects and 64 from the patients. The firing rates were lower in patients' (4.6–14.3/s, mean 7.9/s) than in control subjects' MUs (5.4–20/s, mean 10.6/s). The difference was highly statistically significant ($p < 10^{-6}$). Patient's MU firing was often irregular, and single MUs could not always be followed through the recordings of an entire experiment, presumably due to fatigue. Therefore, some MUs could not be taken for variability analysis after recognition due to insufficient number of points. The slow fluctuations of MU firing rate were also much more pronounced in patients than in control subjects.

Figure 2 illustrates the differences in pattern of MU discharge between control subjects and patients. The upper panels of Figs. 2(a) and 2(b) present typical plots of instantaneous firing rate vs. time of discharge for a control subject and a patient, respectively. Instantaneous firing rate was calculated as the reciprocal of moving average, computed over 7 consecutive ISIs, and time of discharge corresponded to the middle discharge of these ISIs. The figure thus shows the slow variability of the firing rates, which is known to be correlated with the fluctuations in muscle force [34]. Control subjects were able to keep steady muscle contraction, so the slow variability of firing rate was usually limited to small fluctuations around a constant value, presumably resulting from reflex force regulation processes. Therefore, joint interval histograms for control subjects (Fig. 2(a), lower panel) usually did not exhibit any visible trends (serial correlation coefficient around 0). The patients were unlikely to control the muscle force effectively, hence their firing rates fluctuated more slowly and were with much higher amplitude than those of control subjects. In line with the result of the visible trends (where short intervals were likely to be followed by short ones and long intervals by long ones), joint interval histograms for spastic patients were usually positive (Fig. 2(b), lower panel). The same phenomenon, however, could be observed in control MUs over the rare fragments with trends (Fig. 2(c)). Similarly, when a relatively stationary fragment could be found in spastic MU firing, the joint histogram did not reveal any positivity (Fig. 2(d)). It should be stressed that patterns presented in Figs. 2(c) and 2(d) were much more scarce than those shown in Figs. 2(a) and 2(b).

Complete CD_m(*MISI*) relationships were obtained for 25 control MUs and 37 MUs from 10 patients. One patient's MU firing was too irregular to be taken for further analysis. Figure 3 shows an example of CD2-MISI2 relationship from a control subject (small circles). Big diamonds are the mean values (*CDM*) calculated by averaging *CD2* values falling into the vertical bins of 10-ms width (moved every 5 ms) and plotted against *MISI*, determined as the central ISI value in the bin. The *CDM* values calculated from too small a number of points (peripheral values outside broken lines) are neglected. The intersection of the two lines fitted to the linear fragments of remaining *CDM*(*MISI*) plot (as described in the Methods section) determines the value of the TI.

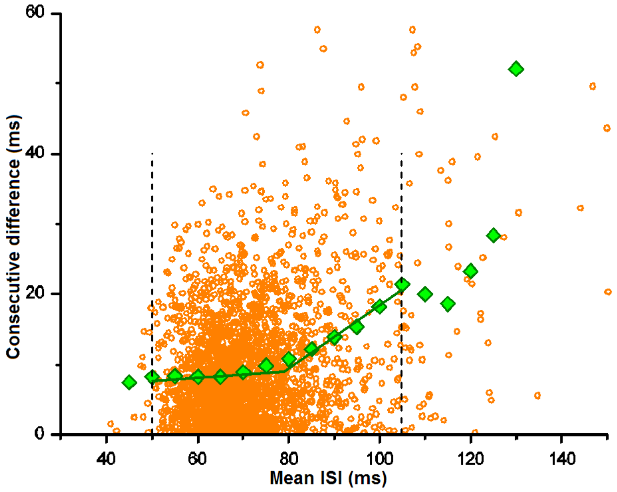

Figure 3. Explanation of the construction of CDM(MISI) plots. Small open circles: CD2(MISI2); diamonds: CDM(MISI). Solid line was determined by modified least-squares procedures, as described in Methods, from the points inside the reliability limits (broken vertical lines). Transition interval was determined as the intersection of the least-squares lines.

Figure 4 shows $CD_m(MISI)$ relationships for all MUs of spastic patients (diamonds) and pooled control data (dots). The $CD_m(MISI)$ relationships of patients were shifted towards longer ISIs. Note that patients' ISI variability was visibly lower than the variability of control MUs. This difference was statistically significant ($p < 0.001$). However, when the variability in the short-interval range was compared between patients and control subjects (meaning that for each MU, only these SD values were selected, which corresponded to mean ISIs shorter than TI), the difference was not statistically significant.

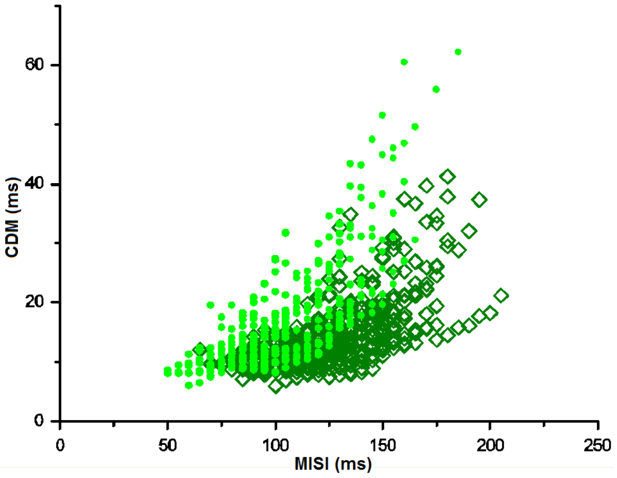

Figure 4. Pooled plot CDM(MISI). Filled small circles: control subjects. Open diamonds: spastic patients.

In Fig. 5, mean values of TIs (calculated for each subject from all MUs analyzed) are plotted vs. age for control subjects (circles) and spastic patients (triangles). The patients' TIs were substantially longer than control values. Moreover, their dependencies on age differed from each other. The regression equations were $y = 0.53x + 78.952$ (regression coefficient $R = 0.692$) for the control group and $y = -0.324x + 151.56$ ($R = 0.342$) for the patient group. This difference was statistically significant ($p < 0.005$). Note that the patients' regression line tends to converge with the control one. We correlated also the increase in TI (calculated with respect to the control regression line) with the duration of the disorder (Fig. 6). The regression was insignificant, when all points were considered ($R = 0.377$, asterisks). However, when the data of 3 patients older than 55 years were removed, the significance substantially increased ($R = 0.856$, circles).

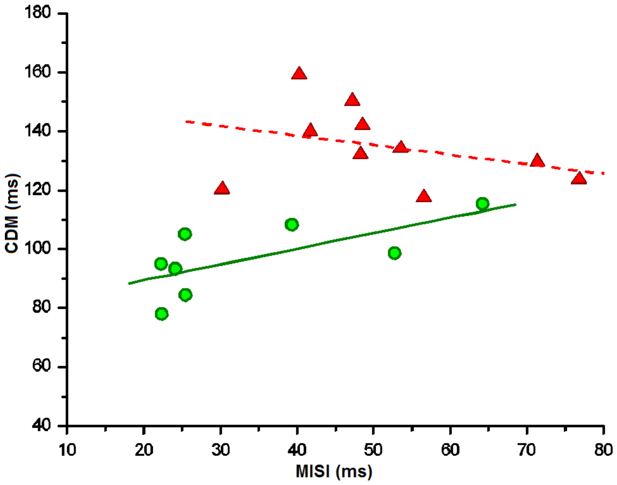

Figure 5. Mean values of transition interval calculated for each subject from single MU data: circles, solid line, control subjects; triangles, broken line, spastic patients.

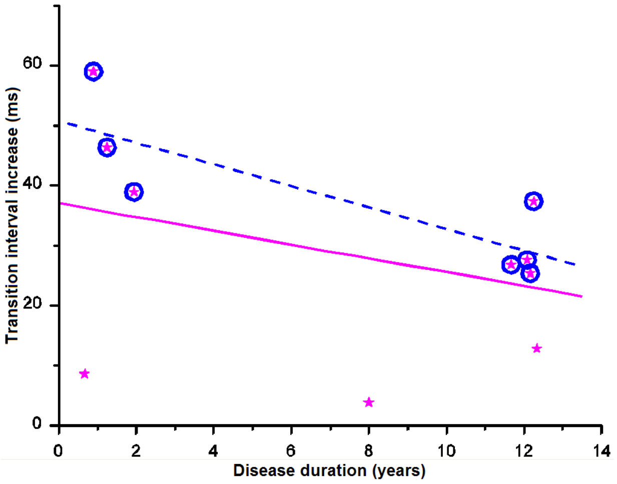

Figure 6. Correlation between transition interval increase and disease duration: asterisks, solid line, pooled data; open circles, broken line, data of patients younger than 55 years.

4. Discussion

Our results concerning MU discharge characteristics conform in general to those of previous reports, showing positive serial correlation coefficients [6,18], lesser ISI variability [18], and lower MU firing rates [4,17,18] in patients compared with control subjects. However, some of these findings need additional comment.

It has been shown here that the positivity observed for serial correlation coefficient in most spastic MUs is due to the trends in MU firing rates related to less efficient control of the muscle force.

The results of comparison of ISI variability between different subject groups are very much influenced by the method applied. As is evident (cf. Fig. 3), the variability strongly depends on the mean ISI. This dependency is a consequence of the difference in spike generating modes in short- and long-interval ranges. The short-interval range corresponds to the *rhythmic firing mode* [35] when each consecutive spike is initiated as the result of the firing threshold crossing by the linearly rising membrane potential trajectory. In this mode, MN discharges are regular, their distribution is close to normal, and the ISI variability is low. The long-interval range (sub-primary range [36]) corresponds to the *occasional spike mode* [35] when a large fraction of ISIs is longer than the AHP of the MN. In the final part of these ISIs, the equilibrium membrane potential lies below threshold and the MN is being randomly excited by synaptic noise [35,37]. In this firing mode, MN discharge is irregular, ISI distribution has an exponential tail of long intervals, and the variability increases rapidly with the mean ISI.

The most reasonable way to assess differences in variability is to make the comparison with the same mode of spike generation. From the two modes described above, the one with rhythmic firing seems to be more appropriate, since the set of variability values here would be less dependent on the firing rate range, at which the data was collected. Moreover, the upper end of ISI duration (corresponding to the highest variability) is the least reliable, since it is affected by the subject's ability to keep steady contraction at the lowest contraction levels. The comparison of the short-interval variability yielded no difference between control and patient group. It may be expected that the lower variability in spastic MUs reported by Andreassen and Rosenfalck [18] was also due to the shift of variability-mean dependency toward longer intervals. Results similar to ours were yielded by a study of Sun et al. [7], in which short-term variability was investigated separately from long-term variability (related to trends). Short-term variability in stroke patients was within control limits, whereas long-term variability was significantly higher.

The TI computed for single MUs has been shown to correlate with AHP of their MNs [28,31]. Thus, our results indicate that MNs of spastic patients become "slower" than those of control subjects. This explains commonly observed decrease in firing rates, since they are known to be controlled by MN AHP. This also suggests that the match between MN and muscle properties is preserved in post-stroke patients.

The approximation lines for control subjects and patients tend to converge, which means that the susceptibility to changes due to the cerebral lesion decreases with age. This is with no doubt a consequence of the age-dependent decrease in MN plasticity. However, there is also another factor influencing AHP duration. The increase in MN AHP duration (expressed by TI) for patients younger than 55 years was strongly correlated with disease duration, which implies that MN properties were gradually recovered with time after the stroke accident. The recovery was less pronounced in older patients, which also may be attributed to the decreased MN plasticity. This issue, however, calls for further investigation on a bigger patient sample with wider range of disease durations.

5. Conclusion

Our data indicated that the spinal MNs responded to the cerebral stroke with prolongation of AHP duration, which tended to recover after the accident. These changes were less pronounced in older patients, presumably due to decreased MN plasticity. We conclude that the match between MN and muscle properties is preserved after stroke. More studies are necessary to verify these conclusions based on the preliminary data.

Acknowledgements

The authors are indebted to Dr. Adam Jozwik for his help with statistical data analysis. This work was supported by the Personnel Exchange Program between the National Scientific Council, Taiwan and Polish Academy of Sciences (research topic: Investigation of Peripheral Neuromuscular System Involvement in CNS lesions).

References

[1] V. Dietz, U. P. Ketelsen, W. Berger and J. Quintern, "Motor unit involvement in spastic paresis. Relationship between leg muscle activation and histochemistry," *J. Neurol. Sci.*, 75: 89-103, 1986.

[2] N. J. Davey, P. H. Ellaway, C. L. Friedland and D. J. Short, "Motor unit discharge characteristics and short term synchrony in paraplegic humans," *J. Neurol. Neurosurg. Psychiatry*, 53: 764-769, 1990.

[3] W. R. Frontera, L. Grimby and L. Larsson, "Firing rate of the lower motoneuron and contractile properties of its muscle fibers after upper motoneuron lesion in man," *Muscle Nerve*, 20: 938-947, 1997.

[4] J. J. Gemperline, S. Allen, D. Walk and W. Z. Rymer, "Characteristics of motor unit discharge in subjects with hemiparesis," *Muscle Nerve*, 18: 1101-1114, 1995.

[5] A. Rosenfalck and S. Andreassen, "Impaired regulation of force and firing pattern of single motor units in patients with spasticity," *J. Neurol. Neurosurg. Psychiatry*, 43: 907-916, 1980.

[6] B. T. Shahani, M. M. Wierzbicka and S. W. Parker, "Abnormal single motor unit behavior in the upper motor neuron syndrome," *Muscle Nerve*, 14: 64-69, 1991.

[7] T. Y. Sun, J. J. Chen and T. S. Lin, "Analysis of motor unit firing patterns in patients with central or peripheral lesions using singular-value decomposition," *Muscle Nerve*, 23: 1057-1068, 2000.

[8] O. Eerbeek, D. Kernell and B. A. Verhey, "Effects of fast and slow patterns of tonic long-term stimulation on contractile properties of fast muscle in the cat," *J. Physiol.-London*, 352: 73-90, 1984.

[9] D. Kernell, O. Eerbeek, B. A. Verhey and Y. Donselaar, "Effects of physiological amounts of high- and low-rate chronic stimulation on fast-twitch muscle of the cat hindlimb. I. Speed- and force-related properties," *J. Neurophysiol.*, 58: 598-613, 1987.

[10] D. Pette and G. Vrbová, "What does chronic electrical

stimulation teach us about muscle plasticity?" *Muscle Nerve*, 22: 666-677, 1999.

[11] S. Salmons and G. Vrbová, "The influence of activity on some contractile characteristics of mammalian fast and slow muscles," *J. Physiol.-London*, 201: 535-549, 1969.

[12] R. R. Roy, R. D. Sacks, K. M. Baldwin, M. Short and V. R. Edgerton, "Interrelationships of contraction time, Vmax, and myosin ATPase after spinal transection," *J. Appl. Physiol.*, 56: 1594-1601, 1984.

[13] G. Grimby, C. Broberg, I. Krotkiewska and M. Krotkiewski, "Muscle fiber composition in patients with traumatic cord lesion," *Scand. J. Rehabil. Med.*, 8: 37-42, 1976.

[14] T. C. Cope, S. C. Bodine, M. Fournier and V. R. Edgerton, "Soleus motor units in chronic spinal transected cats: physiological and morphological alterations," *J. Neurophysiol.*, 55: 1202-1220, 1986.

[15] J. Celichowski, W. Mrowczynski, P. Krutki, T. Gorska, H. Majczynski and U. Slawinska, "Changes in contractile properties of motor units of the rat medial gastrocnemius muscle after spinal cord transection," *Exp. Physiol.*, 91: 887-895, 2006.

[16] C. K. Thomas, "Contractile properties of human thenar muscles paralyzed by spinal cord injury," *Muscle Nerve*, 20: 788-799, 1997.

[17] J. L. Young and R. F. Mayer, "Physiological alterations of motor units in hemiplegia," *J. Neurol. Sci.*, 54: 401-412, 1982.

[18] S. Andreassen and A. Rosenfalck, "Impaired regulation of the firing pattern of single motor units," *Muscle Nerve*, 1: 416-418, 1978.

[19] M. Piotrkiewicz, L. Kudina, J. Mierzejewska, M. Jakubiec and I. Hausmanowa-Petrusewicz, "Age-related change in duration of afterhyperpolarization of human motoneurones," *J. Physiol.-London*, 585: 483-490, 2007.

[20] L. Mazurkiewicz and M. Piotrkiewicz, "Computer system for identification and analysis of motor unit potential trains," *Biocybern. Biomed. Eng.*, 24: 15-23, 2004.

[21] T. Tokizane and H. Shimazu, *Functional Differentiation of Human Skeletal Muscle*, Springfield: Charles C. Thomas, 1964.

[22] J. Hannerz, "Discharge properties of motor units in relation to recruitment order in voluntary contraction," *Acta Physiol. Scand.*, 91: 374-385, 1974.

[23] T. K. Khuskivadze, "Comparison of motor unit firing in gastrocnemius and soleus muscles," *Fiziol Čeloveka (Moscow)*, 5: 102-109, 1979. [Russian]

[24] H. Kranz and G. Baumgartner, "Human alpha motoneurone discharge, a statistical analysis," *Brain Res.*, 67: 324-329, 1974.

[25] B. Maton, "Fréquence et recrutement des unités motrices du muscle biceps brachial au cours du travail statique chez l'homme normal," *J. Physiol.-Paris*, 73: 177-199, 1977.

[26] R. S. Person and L. P. Kudina, "Discharge frequency and discharge pattern of human motor units during voluntary contraction of muscle," *Electroencephalogr. Clin. Neurophysiol.*, 32: 471-483, 1972.

[27] R. S. Person, "Spinal mechanisms of muscle contraction control," in: T. M. Turpaev (Ed.), *Soviet Scientific Review Series, Section F*, New Jersey: Harwood Academic Publishers, 1-83, 1993.

[28] M. Piotrkiewicz, "An influence of afterhyperpolarization on the pattern of motoneuronal rhythmic activity," *J. Physiol.-Paris*, 93: 125-133, 1999.

[29] M. Piotrkiewicz, L. Kudina, I. Hausmanowa-Petrusewicz, N. Zhoukovskaya and J. Mierzejewska, "Discharge properties and afterhyperpolarization of human motoneurons," *Biocybern. Biomed. Eng.*, 21: 53-75, 2001.

[30] M. Piotrkiewicz, "Modelling of motoneuronal rhythmic activity," *Biocybern. Biomed. Eng.*, 20: 35-43, 2000.

[31] R. K. Powers and M. D. Binder, "Relationship between the time course of the afterhyperpolarization and discharge variability in cat spinal motoneurones," *J. Physiol.-London*, 528: 131-150, 2000.

[32] G. R. Holt, W. R. Softky, C. Koch and R. J. Douglas, "Comparison of discharge variability in vitro and in vivo in cat visual cortex neurons," *J. Neurophysiol.*, 75: 1806-1814, 1996.

[33] M. Piotrkiewicz, I. Hausmanowa-Petrusewicz, J. Mierzejewska and M. Jakubiec, "Motoneuron 'fastness' in amyotrophic lateral sclerosis," in: G. Vrbova, M. Piotrkiewicz and W. Zmyslowski (Eds.), *Lecture Notes of 78th ICB Seminar 'Motoneurons and Motoneuron Pools'*, Warsaw: ICB, 69-76, 2005.

[34] C. J. De Luca and Z. Erim, "Common drive of motor units in regulation of muscle force," *Trends Neurosci.*, 17: 299-305, 1994.

[35] W. H. Calvin, "Three modes of repetitive firing and the role of threshold time course between spikes," *Brain Res.*, 69: 341-346, 1974.

[36] L. P. Kudina, "Analysis of firing behaviour of human motoneurones within 'subprimary range'," *J. Physiol.-Paris*, 93: 115-123, 1999.

[37] P. B. Matthews, "Properties of human motoneurones and their synaptic noise deduced from motor unit recordings with the aid of computer modelling," *J. Physiol.-Paris*, 93: 135-145, 1999.
